# Sequential Application of the Birmingham Atypical Cartilage Tumor Imaging Protocol and Radiological Aggressiveness Score in Long-Bone Intramedullary Cartilage Tumors: A Retrospective Cohort Study

**DOI:** 10.3390/diagnostics16213583

**Published:** 2026-10-30

**Authors:** Caleb C. Cummings, Joseph Redding, Chloe Chose, Sarah C. Tepper, David M. Joyce, Odion T. Binitie, Jamie T. Caracciolo, Alexander L. Lazarides

**Affiliations:** 1 USF Health Morsani College of Medicine, University of South Florida, 560 Channelside Drive, Tampa, FL 33602, USA; calebcummings@usf.edu (C.C.C.); jtredding@usf.edu (J.R.); cchose@usf.edu (C.C.); 2 Department of Sarcoma, Moffitt Cancer Center, 12920 USF Magnolia Drive, Tampa, FL 33612, USA; sarah.tepper@moffitt.org (S.C.T.); david.joyce@moffitt.org (D.M.J.); odion.binitie@moffitt.org (O.T.B.); 3 Department of Musculoskeletal Radiology, Moffitt Cancer Center, 12920 USF Magnolia Drive, Tampa, FL 33612, USA; jamie.caracciolo@moffitt.org

**Keywords:** BACTIP, chondrosarcoma, intramedullary cartilage tumor, radiological aggressiveness score, referral triage

## Abstract

**Background**: The Birmingham Atypical Cartilage Tumor Imaging Protocol (BACTIP) addresses referral triage, whereas the Radiological Aggressiveness Score (RAS) estimates resection-grade histology. We evaluated their sequential application in a selected tertiary-center cohort. **Methods**: This retrospective study included 99 patients during 2001–2025, combining consecutive eligible surgical chondrosarcomas with a nonconsecutive enchondroma sample. Groups comprised lesions outside BACTIP referral categories, referral-category lesions with RAS < 4, and referral-category lesions with RAS ≥ 4. Diagnostic performance for resection-grade disease (grade 2, grade 3, or dedifferentiated chondrosarcoma) and exploratory, unadjusted oncologic outcomes were assessed. **Results**: All 47 resection-grade tumors met BACTIP referral criteria (sensitivity 100%; 95% CI, 92.4–100%). Requiring RAS ≥ 4 increased whole-cohort specificity from 69.2% to 94.2% and reduced sensitivity to 91.5% (95% CI, 80.1–96.6%). RAS positivity alone and sequential classification identified identical patients. Four grade 2 tumors were RAS-negative despite BACTIP category 3 classification, and grade 2 sensitivity was 78.9% (15/19). Excluding dedifferentiated tumors, sensitivity was 83.3% (20/24). Oncologic events were concentrated in the referral/RAS-positive group, and global log-rank comparisons were significant for all three endpoints (*p* < 0.001). **Conclusions**: BACTIP may structure referral assessment, while RAS may further characterize imaging aggressiveness in lesions meeting referral criteria. The four RAS-negative grade 2 tumors underscore the importance of maintaining specialist referral when BACTIP referral criteria are met, even when RAS suggests lower imaging aggressiveness. Neither tool replaces clinical assessment, histopathological correlation, or multidisciplinary review. This selected cohort does not establish safe nonreferral, independent prognostic value, or improved management or outcomes. Prospective validation in the intended referral population is required.

## 1. Introduction

Chondrosarcoma is a common primary bone sarcoma in adults, and treatment varies based on tumor grade and aggressiveness. Intermediate and high grade chondrosarcomas (grade 2, grade 3, and dedifferentiated) are classified as resection-grade, due to the elevated risks of metastasis and mortality in this group [1], which, when paired with chondrosarcoma’s underlying chemo- and radio resistance, makes surgery the mainstay of treatment [2,3,4,5]. Conversely, grade 1 tumors have more variation in treatment aggressiveness, typically between curettage with adjuvant therapy, monitoring, or resection, with very low rates of metastasis (0–6%) [6,7], especially in lesions primary to the appendicular skeleton (approaching 0%) [8]. Finally, enchondromas are benign chondroid tumors that are typically monitored radiologically if asymptomatic, because malignant transformation is uncommon [9,10].

Even so, accurate, reliable preoperative characterization of lesions to inform treatment decision-making remains a challenge. Undergrading may lead to intralesional curettage of a tumor for which wide resection would ordinarily be considered, risking inadequate local control and further surgery; overgrading may expose patients to unnecessary resection and reconstruction. Diagnosis has long relied on correlation of histopathology with clinical and radiographic findings. Because biopsy samples only part of a potentially heterogeneous lesion, imaging remains essential to its interpretation [10,11]. Structured imaging tools address different stages of this assessment, from referral triage to characterization of tumor aggressiveness. The Birmingham Atypical Cartilage Tumor Imaging Protocol (BACTIP) was developed as a structured imaging-based triage framework intended to assist non-specialist musculoskeletal clinicians in determining whether cartilage lesions of the proximal humerus and around the knee should be discharged, monitored radiographically, or referred for specialist oncologic evaluation [12]. Another imaging tool used in the characterization of long-bone intramedullary chondroid lesions is the Radiological Aggressiveness Score (RAS). This score estimates the likelihood of resection-grade histology after specialist referral and may supplement preoperative assessment. The RAS is made up of nine imaging parameters, and the presence of 4 or more parameters predicts resection-grade histology (grade 2, 3 or dedifferentiated chondrosarcoma) [11].

BACTIP and RAS address related but different questions: referral triage and the likelihood of resection-grade histology. The contribution of this study is a retrospective description of how referral categories and an aggressiveness score intersect in an existing tertiary-center cohort, including clinically relevant discordant classifications. RAS is considered here as an aid to estimating resection-grade disease after referral, not as a validated means of determining referral urgency or selecting treatment. Therefore, we asked: (1) How were resection-grade lesions distributed across BACTIP referral categories in this tertiary-center cohort? (2) Did RAS further stratify histologic aggressiveness among lesions meeting BACTIP referral criteria? (3) How were observed oncologic events distributed across sequential BACTIP-RAS groups?

## 2. Materials and Methods

### 2.1. Study Design and Setting

We performed a retrospective cohort study of patients presenting to a single tertiary orthopaedic oncology center with an intramedullary cartilage tumor of a long bone over a 24-year period. Chondrosarcoma cases were identified from an institutional database of surgically treated patients, and enchondroma cases were sampled separately from institutional records and the imaging archive. Both tools were applied retrospectively, thus the sequential classification did not direct the patients’ referral or treatment.

### 2.2. Patients

**Figure 1:**
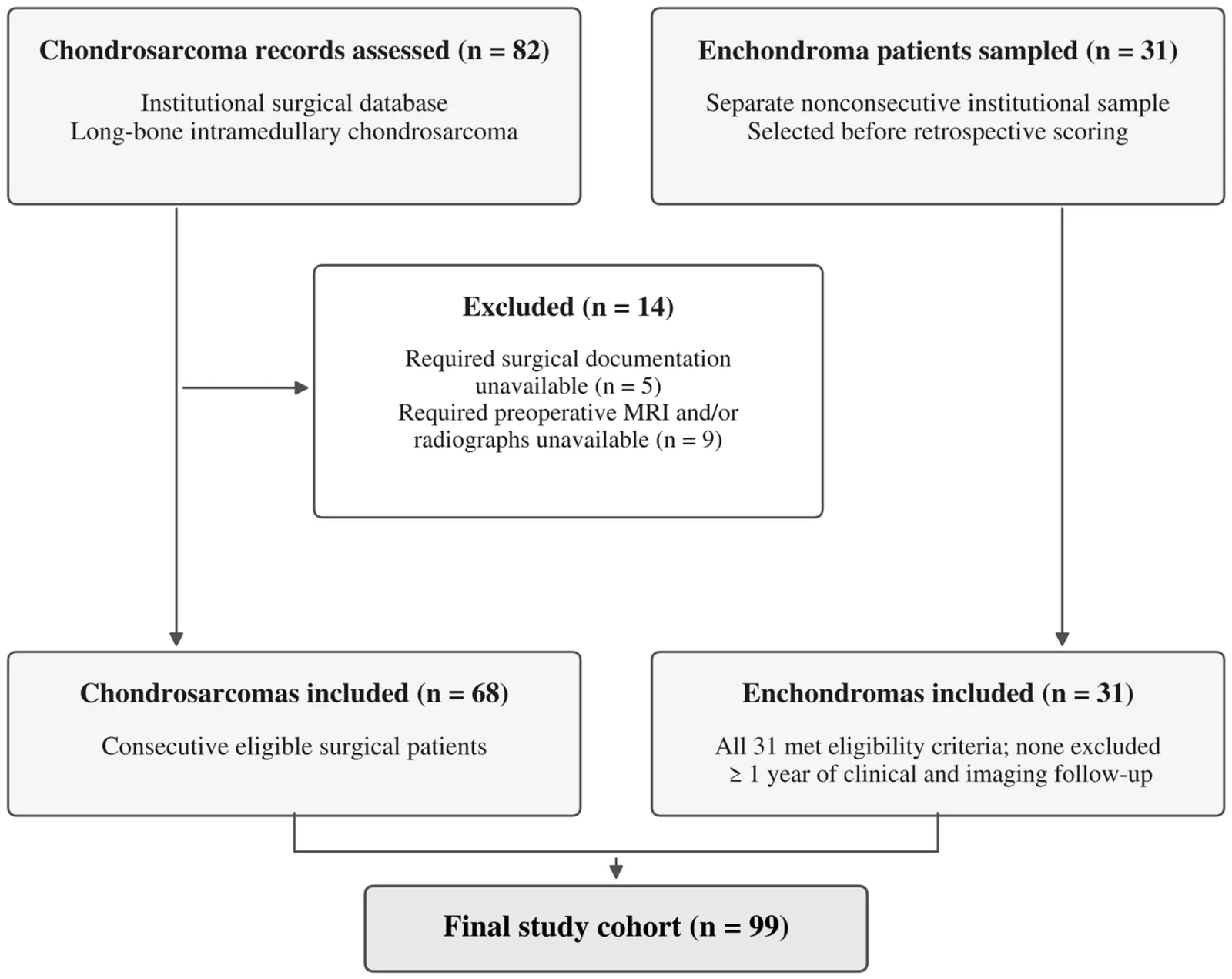
Retrospectively reconstructed patient selection. Chondrosarcoma records were identified from a surgical database. Enchondroma patients were sampled separately and did not require surgery, and all sampled enchondroma patients met inclusion criteria.

The cohort comprised 99 patients with long-bone intramedullary cartilage tumors evaluated between January 2001 and May 2025. Eligible patients with surgically managed, histologically confirmed chondrosarcoma were included consecutively. Retrospective reconstruction of patient selection identified 82 surgical database records, of which 14 were excluded because required surgical documentation (n = 5) or preoperative MRI and/or radiographs (n = 9) were unavailable, leaving 68 chondrosarcomas. A separate, nonconsecutive sample of 31 institutional patients with enchondroma was selected before retrospective BACTIP and RAS scoring. All met the inclusion criteria, including at least one year of clinical and imaging follow-up, and were included (Figure 1). Surgery was not required for the enchondroma group. All included patients had diagnostic radiographs and MRI of sufficient quality for classification. There was no patient overlap with the RAS derivation cohort of Gundavda et al. [11], which was assembled at a separate institution.

Reference diagnoses were established by surgical histopathology in operated patients, or by the treating oncologists and radiologists using clinical and radiological presentation and subsequent follow-up in nonsurgical patients. None of the nonsurgical patients underwent biopsy, and inclusion required at least one year of clinical and imaging follow-up. Imaging stability was assessed retrospectively and defined as an unchanged BACTIP category on serial MRI and radiographs. Diagnoses were categorized as enchondroma, grade 1, grade 2, grade 3, or dedifferentiated chondrosarcoma. Resection-grade disease comprised grade 2, grade 3, and dedifferentiated chondrosarcoma, consistent with the grade-based endpoint of the original RAS study [11].

### 2.3. BACTIP and RAS Classification

Two medical student reviewers (C.C.C. and J.R.) received supervised training in musculoskeletal tumor imaging from an orthopaedic oncologic surgeon (A.L.L.) and a musculoskeletal radiologist (J.T.C.) before study image review. They jointly evaluated all available imaging, including follow-up examinations, and assigned BACTIP categories and RAS by consensus. Cases requiring additional input were discussed with an orthopaedic oncologic surgeon or musculoskeletal radiologist to establish a final classification. Baseline scoring was blinded to radiology reports, clinic notes, pathology, treatment, and outcomes. All imaging studies were reviewed using Centricity Universal Viewer (GE HealthCare, Chicago, IL, USA). Baseline scores used the latest available preoperative studies for surgically managed patients and the first presenting studies for nonsurgically managed patients. Imaging-to-reference-diagnosis intervals were not systematically recorded, including imaging-to-surgery intervals. Scanner models, field strengths, acquisition parameters, and MRI sequence availability were not systematically recorded during review, so protocol consistency could not be established. Joint consensus scores did not permit interobserver or intraobserver reliability assessment.

BACTIP classification was based on lesion size, degree of endosteal scalloping, and the presence of aggressive imaging features. Lesions were assigned to class 1 (<4 cm) or class 2 (≥4 cm) and further subdivided according to endosteal scalloping as type A (none), type B (focal endosteal scalloping involving <10% of the circumference, equivalent to an angle <36°), or type C (generalized scalloping). Lesions demonstrating bone expansion, cortical thickening, periostitis, cortical destruction, or a soft-tissue mass were classified as category 3. Category 1A was designated as an immediate-discharge category, categories 2A, 1B, and 2B as surveillance categories, and categories 1C, 2C, and 3 as referral categories, in accordance with the BACTIP management framework [12].

Radiological aggressiveness was assessed using the nine-parameter RAS described by Gundavda et al. [11]. The score incorporates radiographic and MRI features associated with increased biologic aggressiveness, including endosteal scalloping of >2/3 axial lesion circumference, endosteal scalloping of >2/3 lesion length, medullary expansion, cortical breakthrough, periosteal reaction, pathological fracture, cortical thickening, marrow edema, and soft-tissue extension. One point was assigned for the presence of each parameter, yielding a total score ranging from 0 to 9. Consistent with the original RAS derivation study, a score of ≥4 was considered positive and indicative of an increased likelihood of resection-grade disease [11].

Patients were stratified into three groups: lesions not meeting BACTIP referral criteria (categories 1A, 1B, 2A, and 2B), lesions meeting referral criteria (categories 1C, 2C and 3) with RAS < 4, and lesions meeting referral criteria with RAS ≥ 4. Because all patients had already presented to a tertiary orthopaedic oncology center, BACTIP referral status was assigned retrospectively. Accordingly, the group of lesions not meeting referral criteria represents lesions that would not have met referral criteria under the protocol, thus it does not represent a community cohort of patients who were actually discharged or never referred. Lesions in BACTIP categories 1C, 2C, and 3 met referral criteria regardless of RAS score, then RAS provided subsequent stratification of imaging aggressiveness.

### 2.4. Outcome Measures

The primary outcome was the diagnostic performance of the sequential BACTIP-RAS classification for identifying resection-grade disease. For BACTIP alone, a positive classification meant meeting referral criteria. For RAS alone, a positive result meant RAS ≥ 4. For the sequential classification, a positive result required both BACTIP referral criteria and RAS ≥ 4. Sensitivity, specificity, positive predictive value, negative predictive value, and overall accuracy were reported for these classifications across the full cohort. Additional analyses examined sensitivity by histologic grade, performance after exclusion of dedifferentiated chondrosarcoma, performance restricted to surgical histopathology, and performance restricted to original BACTIP sites (proximal humerus, distal femur, or proximal tibia). The endpoint was resection-grade diagnosis, not the clinical necessity of referral or surgery.

Secondary outcomes were local recurrence, new metastasis, final disease status, recurrence-free survival, metastasis-free survival, and disease-specific survival. Baseline metastases were reported separately. Final status comprised alive with no evidence of disease (ANED), alive with disease (AWD), died of disease (DOD), or death from another cause. Time was measured from surgery in operated patients and initial imaging in nonsurgical patients to the event or last recorded status. Recurrence-free survival was defined as freedom from first local recurrence or distant metastatic progression, and metastasis-free survival measured time to first distant relapse, including distant relapse after an earlier local recurrence. The three patients metastatic at presentation were excluded from both analyses (n = 96). Disease-specific survival included all 99 patients, with tumor-related deaths as events and confirmed deaths from other causes censored at death. For recurrence and metastasis endpoints, death without the relevant event was censored at death. Remaining patients were censored at their last recorded status date.

### 2.5. Statistical Analysis

Continuous baseline variables were summarized as medians and interquartile ranges and compared using global Kruskal–Wallis tests. Sex and pain comparisons used two-sided Fisher–Freeman–Halton exact tests, and larger categorical tables used a Monte Carlo approximation with 99,999 samples (random seed 20261025). Diagnostic performance was calculated from two-by-two counts with two-sided 95% Wilson score confidence intervals, without continuity correction. Thresholds were not refitted. Exploratory temporal comparisons used broad calendar periods of 2001–2012 and 2013–May 2025, assigned by surgery date in operated patients or initial imaging date in nonsurgical patients. Sensitivity and specificity were compared using two-sided Fisher exact tests. Exploratory Firth bias-reduced logistic regression modeled resection-grade disease using RAS positivity, age at imaging per 10 years, and sex. Adjusted odds ratios, profile penalized-likelihood 95% confidence intervals, and penalized likelihood-ratio *p*-values were reported. Firth estimation was used to reduce small-sample bias given the strong RAS association, and conventional maximum-likelihood estimates were examined as a sensitivity check. Because RAS and sequential positivity were identical, they were not entered together. Kaplan–Meier estimates used pointwise Greenwood log–log confidence intervals and global three-group log-rank tests, not pairwise comparisons. Reverse Kaplan–Meier follow-up treated death from any cause as censored and observation ending alive as an event. Separately, nonsurgical imaging follow-up was the interval from initial imaging diagnosis to the latest recorded follow-up imaging examination, summarized using ordinary medians and interquartile ranges. Oncologic comparisons remained exploratory and unadjusted because event-free and sparse-event groups prevented reliable conventional multivariable group comparisons. All comparisons and additional analyses were exploratory, with *p* < 0.05 used descriptively and no multiplicity adjustment. Analyses used Python (version 3.13, Python Software Foundation), NumPy, pandas, SciPy, and Matplotlib, and reproducible code is available upon reasonable request.

## 3. Results

The cohort comprised 99 patients with intramedullary cartilage lesions: 31 enchondromas, 21 grade 1, 19 grade 2, five grade 3, and 23 dedifferentiated chondrosarcomas. Age at imaging, sex, maximum imaging dimension, and documented pain status were available for all patients. Median age was 61 years (IQR, 53.5–70.0), and 58 patients (58.6%) were female. Median maximum lesion dimension was 6.9 cm (IQR, 3.65–11.20). Lesions involved the femur (n = 60), humerus (n = 31), tibia (n = 7), or radius (n = 1). Pain was documented in 85 patients (85.9%). Table 1 summarizes the cohort by sequential classification group.

Six lesions outside retrospective BACTIP referral categories underwent resection, all with grade 1 histology. Available records described local or regional pain in four cases, including coexisting knee osteoarthritis in two, and the other two cases included a painless 14-cm distal femoral lesion and a 19-cm humeral lesion identified incidentally on imaging. Three had preoperative grade 1 biopsy diagnoses. These findings describe the clinical context, but the specific rationale for selecting resection could not be consistently reconstructed. Retrospective BACTIP categories did not direct treatment.

### 3.1. Distribution of Resection-Grade Lesions Across BACTIP Referral Categories

**Figure 2:**
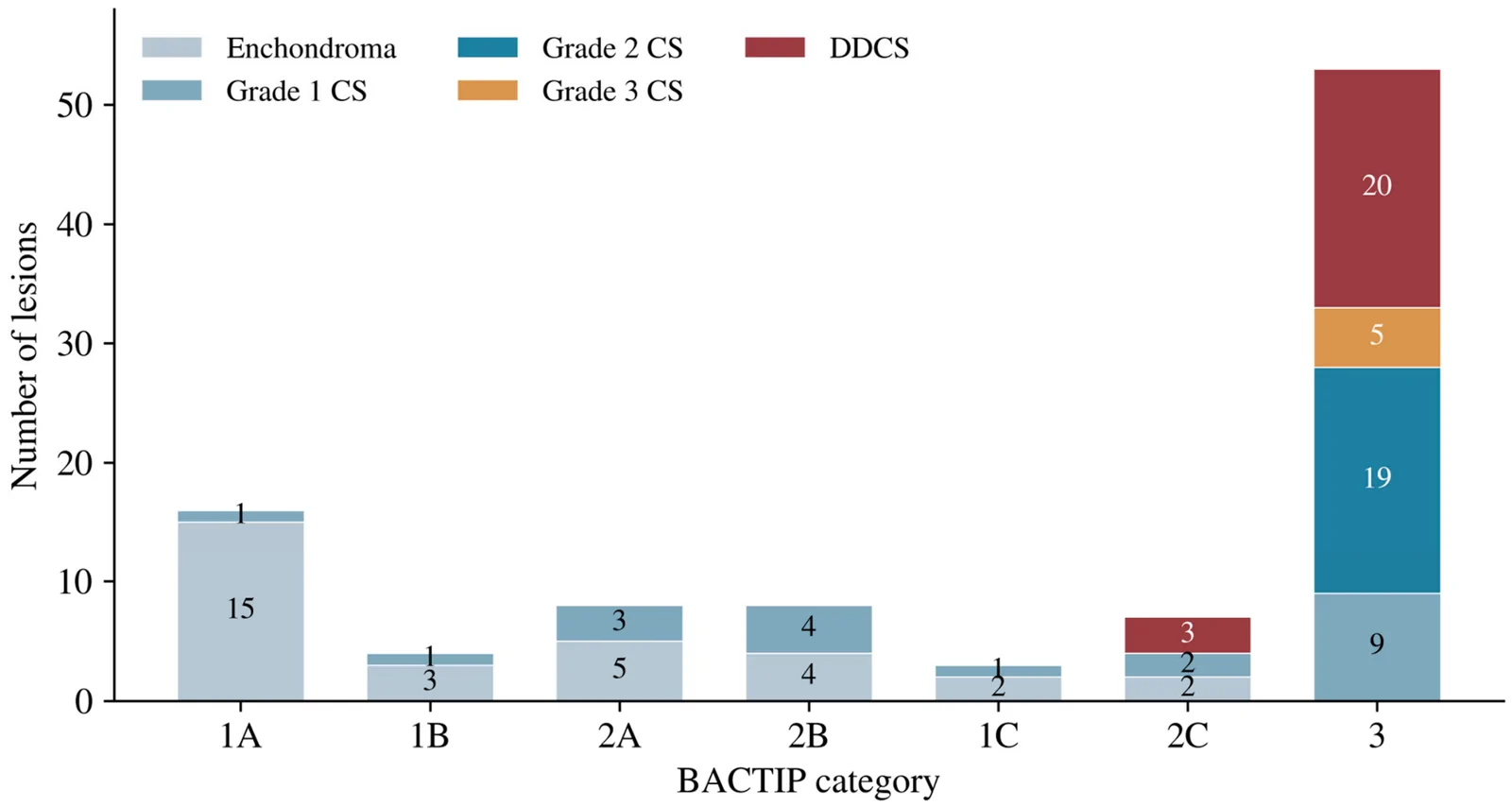
Distribution of reference diagnoses across Birmingham Atypical Cartilage Tumor Imaging Protocol (BACTIP) categories. CS = chondrosarcoma; DDCS = dedifferentiated chondrosarcoma.

Reference diagnosis differed significantly between groups meeting and not meeting BACTIP referral criteria (*p* < 0.001) (Table 1). Most enchondromas were classified within the BACTIP non-referral categories, whereas all resection-grade lesions (grade 2, grade 3, or dedifferentiated chondrosarcoma) were identified within the BACTIP referral categories. Observed surgical management differed between retrospective classification groups (*p* < 0.001), with predominantly nonoperative management among lesions not meeting referral criteria and resection-based treatment among lesions meeting referral criteria (Table 1). Distribution of diagnostic subtype across individual BACTIP categories is shown in Figure 2 and Table 2. Progressive enrichment for aggressive histology was observed with increasing BACTIP category. All resection-grade lesions were confined to BACTIP referral categories 2C and 3, accounting for 43% of category 2C lesions and 83% of category 3 lesions, whereas no enchondromas were classified as category 3.

### 3.2. RAS Stratification Among Lesions Meeting Referral Criteria

Diagnostic performance estimates for the BACTIP referral criteria, RAS alone, and sequential BACTIP-RAS classification for identifying resection-grade lesions are shown in Table 3A–C. In this selected cohort, BACTIP referral categories demonstrated 100% sensitivity (95% CI 92.4% to 100%) and 100% negative predictive value (95% CI 90.4% to 100%) for identifying resection-grade pathology, capturing all grade 2, grade 3, and dedifferentiated chondrosarcomas within the referral categories. Specificity was 69.2% (95% CI 55.7% to 80.1%) because some enchondromas and grade 1 chondrosarcomas also met referral criteria. Requiring both BACTIP referral criteria and RAS ≥ 4 increased whole-cohort specificity to 94.2% (95% CI 84.4% to 98.0%), but reduced sensitivity from 100% to 91.5% (95% CI 80.1% to 96.6%), and positive predictive value was 93.5%. Four of 47 resection-grade lesions (8.5%) were RAS-negative despite meeting BACTIP referral criteria. All were grade 2 chondrosarcomas classified as BACTIP category 3 and underwent surgical resection. Three of these lesions were interpreted as grade 1 chondrosarcoma on preoperative biopsy, whereas the remaining lesion did not undergo biopsy before definitive treatment. These observations describe actual treatment and do not constitute a recommendation to omit biopsy or to select treatment on the basis of RAS alone.

All 46 RAS-positive lesions met BACTIP referral criteria, and none of the 36 lesions outside of referral categories were RAS-positive. RAS alone therefore produced identical classifications and diagnostic performance to sequential BACTIP-RAS classification. Among the 63 lesions meeting referral criteria, RAS specificity was 81.3% (13/16), rather than the whole-cohort value of 94.2%, and negative predictive value was 76.5% (13/17). Sensitivity for grade 2 disease was 78.9% (15/19; 95% CI, 56.7–91.5%). After excluding 23 dedifferentiated tumors, sensitivity was 83.3% (20/24; 95% CI, 64.1–93.3%); specificity remained 94.2% (49/52).

Surgical histopathology established the reference diagnosis in 69 patients (10/36 non-referral, 13/17 referral/RAS-negative, and 46/46 referral/RAS-positive), and the remaining 30 patients (26/4/0) were classified through clinical and imaging follow-up. None of the 30 nonsurgical patients underwent biopsy. In the histology-restricted analysis, sequential/RAS-alone sensitivity was 91.5% (43/47; 95% CI, 80.1–96.6%) and specificity was 86.4% (19/22; 95% CI, 66.7–95.3%). Seventy lesions were located at original BACTIP sites, and 29 were outside those sites. In the 70-patient site-restricted analysis, sensitivity was 91.7% (22/24; 95% CI, 74.2–97.7%) and specificity was 95.7% (44/46; 95% CI, 85.5–98.8%) (Table S1).

The four RAS-negative grade 2 tumors had scores of 2, 3, 2 and 2. None had pathological fracture, scalloping exceeding two-thirds of the axial circumference, cortical breakthrough, or cortical thickening; one had a soft-tissue component. Their category 3 classification reflected other qualifying aggressive features, including expansion, periostitis, or a soft-tissue component. Individual component scores are reported in Table S2; this small series does not establish a specific mechanism or independent predictor of underclassification.

Exploratory temporal analysis showed sequential/RAS sensitivity of 92.0% (23/25; 95% CI, 75.0–97.8%) in 2001–2012 and 90.9% (20/22; 72.2–97.5%) in 2013–2025; specificity was 95.2% (20/21; 77.3–99.2%) and 93.5% (29/31; 79.3–98.2%), respectively (both Fisher *p* = 1.00; Table S5). These results do not establish equivalence across eras. In the age- and sex-adjusted Firth model, RAS positivity remained associated with resection-grade disease (adjusted OR, 106.59; 95% CI, 27.90–595.31; *p* < 0.001). Age per 10 years had an adjusted OR of 1.30 (0.68–2.28; *p* = 0.399), and male sex had an adjusted OR of 4.42 (1.01–25.09; *p* = 0.048). The sex estimate was imprecise and exploratory (Table S6).

### 3.3. Observed Oncologic Events Across Sequential BACTIP-RAS Groups

**Figure 3:**
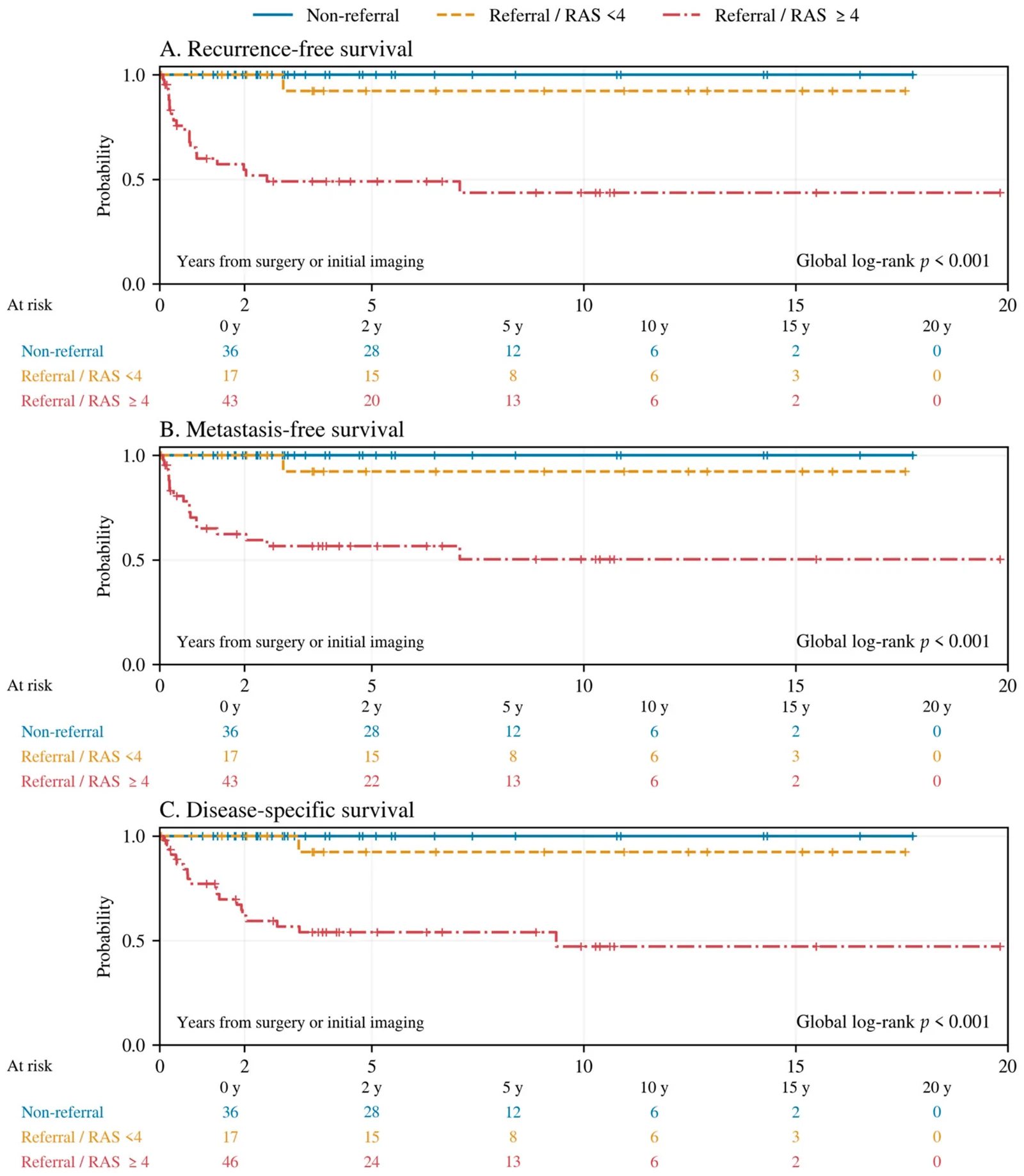
Exploratory Kaplan–Meier estimates by retrospective BACTIP–RAS group: (**A**) recurrence-free survival, defined as freedom from local recurrence or metastatic progression; (**B**) metastasis-free survival; and (**C**) disease-specific survival. Time was measured from surgery in operated patients and initial imaging in nonsurgical patients. Three patients metastatic at presentation were excluded from panels (**A**,**B**) (n = 96; referral/RAS ≥ 4 group n = 43) but retained in panel (**C**) (n = 99; referral/RAS ≥ 4 group n = 46). Death without the relevant event was censored at death, and remaining patients were censored at last recorded status. Plus signs indicate censoring. Tables show numbers at risk. Displayed *p*-values are global three-group log-rank tests. Comparisons are unadjusted and may reflect differences in histological grade, treatment and follow-up.

Observed adverse events were concentrated in the referral/RAS-positive group (Table 4). Three patients had metastases at presentation. Among the 96 patients without baseline metastases, 19 developed new metastases: 18 in the RAS-positive referral group and one in the RAS-negative referral group. All seven local recurrences occurred in the RAS-positive referral group. There were 21 disease-specific deaths (20 RAS-positive and one RAS-negative referral case) and 12 deaths from other causes. No local recurrence, new metastasis, or disease-specific death was recorded in the non-referral group. Global three-group log-rank tests were significant for recurrence-free survival (χ^2^ = 32.49, 2 df), metastasis-free survival (χ^2^ = 25.98, 2 df), and disease-specific survival (χ^2^ = 26.17, 2 df; all *p* < 0.001; Figure 3). These are unadjusted comparisons and do not establish independent prognostic effects.

At five years, estimated recurrence-free, metastasis-free and disease-specific survival in the RAS-positive referral group were 49.0% (95% CI, 32.6–63.5%), 56.6% (39.6–70.5%) and 54.0% (37.5–67.8%), respectively, and 13 patients remained at risk at five years for each endpoint. Corresponding estimates in the RAS-negative referral group were 92.3% (56.6–98.9%; eight at risk) for each endpoint, and no endpoint events were observed in the non-referral group (12 at risk at five years). Reverse Kaplan–Meier median follow-up was 1777 days overall and 1162, 3312 and 2301 days in the non-referral, referral/RAS-negative and referral/RAS-positive groups, respectively. All 30 nonsurgical patients had at least one year of documented imaging and clinical follow-up, with a median of 1221 days (IQR, 850.5–1393; range, 371–5972), equivalent to 3.34 years (IQR, 2.33–3.81). Reference-standard and follow-up details and survival estimates are provided in Tables S3 and S4.

## 4. Discussion

This retrospective cohort illustrates the distinction between identifying lesions that meet BACTIP referral criteria and characterizing imaging aggressiveness after referral. All 47 resection-grade lesions met referral criteria, and 43 of 46 lesions with RAS ≥ 4 were resection-grade. However, four grade 2 tumors had RAS < 4. Because all RAS-positive lesions already met referral criteria, sequential BACTIP-RAS classification and RAS alone had identical diagnostic performance. The contribution is therefore a description of the relationship between the two classifications and their discordant cases, rather than evidence of incremental diagnostic value, improved decisions, or better patient outcomes.

### 4.1. BACTIP Referral Categories

All resection-grade lesions met BACTIP referral criteria in this cohort, and none were identified among categories designated for discharge or surveillance. These findings are consistent with the original intent of BACTIP as a pragmatic referral framework for intramedullary cartilage tumors. The first BACTIP validation study by Davies et al. demonstrated increasing malignancy with higher BACTIP categories and reported only one potentially delayed diagnosis, and a subsequent 780-patient update by Davies et al. demonstrated similarly low rates of delayed diagnosis and malignant transformation [13,14]. External validation studies have provided additional support. In a United States tertiary-care cohort of 292 lesions, Opper et al. reported no cases in which applying BACTIP would have delayed the diagnosis of chondrosarcoma [15]. Van Den Berghe et al. likewise found that aggressive pathology was concentrated within referral categories, with only one grade 2 chondrosarcoma occurring outside the referral categories [16]. Our findings are consistent with concentration of aggressive pathology in higher BACTIP categories. Because BACTIP referral categories include both indolent and aggressive lesions, meeting referral criteria does not establish histologic grade, and further specialist assessment remains necessary.

### 4.2. RAS Stratification

RAS provided additional discrimination among lesions meeting BACTIP referral criteria by identifying a subgroup highly enriched for resection-grade disease. The rationale for using RAS after referral is supported by the original study by Gundavda et al., in which a threshold of four or more radiological aggressiveness features identified resection-grade chondrosarcoma with 97.9% sensitivity and 90.5% specificity [11]. In the present cohort, applying RAS within BACTIP referral categories selected exactly the same lesions as RAS alone. RAS stratification increased whole-cohort specificity from 69.2% to 94.2% at the expense of sensitivity, which fell from 100% to 91.5%. The four resection-grade lesions that lacked RAS positivity were all grade 2 chondrosarcomas classified as BACTIP category 3, and thus still met referral criteria. Three were interpreted as grade 1 chondrosarcoma on preoperative biopsy, while one did not undergo biopsy before definitive treatment. These findings show that RAS < 4 did not exclude resection-grade disease and should not override category 3 features or, by itself, direct discharge, surveillance, or curettage. Rather, concerning imaging that places lesions into BACTIP referral categories warrants continued specialist assessment and radiologic-pathologic review. Thus, a single qualifying category 3 feature could trigger referral even when fewer than four RAS features were present. The component profiles in Table S2 illustrate this threshold difference, but four cases cannot establish which absent features independently explain underclassification.

The specificity–sensitivity trade-off reflects a more restrictive positive definition, not demonstrated clinical utility. BACTIP and RAS also share imaging features: cortical destruction/breakthrough, soft-tissue extension, and endosteal scalloping, although their definitions and thresholds differ. Forty-two of 46 RAS-positive lesions were BACTIP category 3. The tests should therefore not be viewed as independent confirmations of malignancy. After exclusion of dedifferentiated tumors, sensitivity fell to 83.3%, and grade 2 sensitivity was 78.9%. These findings indicate weaker performance in conventional grade 2 disease than the overall estimate suggests. The four discordant category 3 lesions support continued specialist and radiologic-pathologic assessment even when both RAS and a limited biopsy appear less aggressive, though this study did not test a specific escalation or treatment algorithm.

### 4.3. Relationship to Other Risk-Stratification Systems

BACTIP and RAS sit within a broader effort to standardize bone-lesion assessment. BTI-RADS uses cross-sectional imaging for solitary bone tumors, and OT-RADS stratifies osseous tumors using MRI [17,18]. Society of Skeletal Radiology Bone-RADS addresses incidentally detected solitary lesions on CT and MRI, whereas American College of Radiology Bone-RADS and REST provide structured approaches to radiographic risk assessment [19,20,21]. Our analysis is limited to long-bone intramedullary cartilage tumors and did not compare these systems. It therefore does not establish superiority over other frameworks or the need for an additional combined scoring model.

### 4.4. Oncologic Outcomes

Oncologic events occurred predominantly among referral-category lesions with RAS ≥ 4. Although global log-rank tests differed across the three groups, these groups differed markedly in histological grade and treatment, and comparisons used different time origins for operated and nonsurgical patients. The findings may principally reflect underlying disease severity and management rather than an independent prognostic contribution of imaging classification. No local recurrence, new metastasis, or disease-specific death was observed outside referral categories during available follow-up, however, this does not establish the safety of nonreferral or less intensive management. Reverse Kaplan–Meier median follow-up in the non-referral group was 1162 days (IQR, 833–2367), and 8 of 36 patients had observed follow-up shorter than two years. Delayed events in slow-growing lesions remain possible.

### 4.5. Limitations

The principal limitation is referral and spectrum bias: all patients had already presented to a tertiary orthopaedic oncology center, and 47 of 99 had resection-grade disease, including 23 dedifferentiated tumors. This case mix differs substantially from incidentally detected community lesions. Observed sensitivity and specificity depend on the sampled disease spectrum, and predictive values also depend on prevalence. Even if sensitivity and specificity were unchanged, lower prevalence would generally reduce positive predictive value and increase negative predictive value. Additionally, the observed 100% negative predictive value is not proof of safe discharge. Patients missed before referral were not captured, and no comparison with routine multidisciplinary assessment established fewer referrals, changed management, or benefit to patients. The cohort combined consecutive eligible surgical chondrosarcomas with a nonconsecutive enchondroma sample. This sampling strategy limits representativeness.

Secondly, diagnostic verification differed between surgical histopathology and clinical/imaging stability. Stability does not reliably distinguish enchondroma from atypical cartilaginous tumor (ACT), and cannot definitively exclude a slowly progressive lesion. Differential verification and selection for documented stability may misclassify reference diagnoses or preferentially retain indolent lesions, potentially inflating apparent negative predictive value and other performance estimates, and the magnitude and direction of bias cannot be quantified here. Unequal follow-up and events missed after transfer of care further limit interpretation. The histology-restricted sensitivity analysis reduced reference-standard heterogeneity but remained selected by the decision to operate. Although at least one year of clinical and imaging follow-up was required and all included nonsurgical patients had dated imaging follow-up, this does not establish complete attendance at scheduled surveillance visits or exclude events after last contact. Unchanged BACTIP category can also coexist with changes in individual imaging features.

Thirdly, consensus scoring does not establish interobserver or intraobserver reproducibility. Without independent and repeated assessments, neither agreement nor the effect of reader variability on categories, scores, and threshold classifications can be estimated. Joint assessment with specialist input may yield performance that is not reproduced by independent readers or outside specialist practice. The overlapping imaging features of BACTIP and RAS further limit interpretation as independent diagnostic tests.

Fourthly, scanner models, MRI acquisition parameters, and sequence availability were not systematically recorded, preventing assessment of protocol consistency across the 24-year inclusion period. Differences in hardware, resolution, slice thickness, sequences, and image quality could influence feature detection. Changes in diagnostic terminology, biopsy, surveillance, and surgery could also influence selection and outcomes. The exploratory temporal analysis showed similar point estimates, but broad periods, different case mixes, and wide confidence intervals do not establish equivalent performance across eras. Surgery date was a proxy for imaging era in operated patients. Imaging-to-reference intervals were not systematically recorded. BACTIP was developed for the proximal humerus and around the knee [12,13]; 29 lesions were outside those sites. Restriction to 70 explicitly eligible sites yielded sensitivity of 91.7% and specificity of 95.7%, but applicability beyond the original sites remains uncertain.

Finally, the age- and sex-adjusted analysis was exploratory and did not establish demographic-invariant sensitivity or specificity, a validated prediction model, or etiologic associations. Only seven lesions were misclassified by RAS, and the wide adjusted intervals, particularly for sex, limit precision despite bias reduction. No multiplicity adjustment was applied. Oncologic comparisons remain exploratory and unadjusted because classification was strongly associated with grade and treatment, events were sparse in the RAS-negative group and absent in the non-referral group, and time origins differed. These features limit conventional adjusted hazard-ratio estimation. Censoring competing deaths yields cause-specific Kaplan–Meier estimates rather than competing-risk cumulative incidence. Prospective evaluation with standardized imaging and independent readers is needed before claims of clinical utility or safe management.

## 5. Conclusions

In this selected tertiary-center cohort, all 47 resection-grade tumors met BACTIP referral criteria. RAS ≥ 4 identified a subgroup enriched for resection-grade disease. Although requiring both BACTIP referral criteria and RAS ≥ 4 produced the same diagnostic classification as RAS alone, BACTIP referral criteria also captured all four grade 2 tumors with RAS < 4, underscoring that a low RAS score should not override an indication for specialist referral. These observations support further study of BACTIP as a framework for referral assessment and RAS as an adjunct for characterizing imaging aggressiveness after referral. Neither replaces clinical assessment, histopathological correlation, or specialist multidisciplinary review. The approach remains hypothesis-generating and does not establish safe nonreferral, improved treatment selection, independent prognostic value, or better outcomes. Prospective validation in the intended referral population is needed.

## Figures and Tables

**Table 1:** Baseline cohort characteristics stratified by sequential BACTIP-RAS classification.

Characteristic	All Patients (n = 99)	Not Meeting Referral Criteria (n = 36)	Meeting Referral Criteria, RAS < 4 (n = 17)	Meeting Referral Criteria, RAS ≥ 4 (n = 46)	*p*
Age at imaging, years	61.0 (53.5–70.0)	57.0 (51.8–64.3)	61.0 (48.0–70.0)	66.0 (59.0–72.0)	0.016
Female sex	58 (58.6%)	26 (72.2%)	12 (70.6%)	20 (43.5%)	0.020
Reference diagnosis					<0.001
Enchondroma	31 (31.3%)	27 (75.0%)	4 (23.5%)	0 (0.0%)	
Grade 1 chondrosarcoma	21 (21.2%)	9 (25.0%)	9 (52.9%)	3 (6.5%)	
Grade 2 chondrosarcoma	19 (19.2%)	0 (0.0%)	4 (23.5%)	15 (32.6%)	
Grade 3 chondrosarcoma	5 (5.1%)	0 (0.0%)	0 (0.0%)	5 (10.9%)	
Dedifferentiated chondrosarcoma	23 (23.2%)	0 (0.0%)	0 (0.0%)	23 (50.0%)	
Resection-grade disease (grade 2/3/DDCS)	47 (47.5%)	0 (0.0%)	4 (23.5%)	43 (93.5%)	<0.001
Bone location					0.317
Femur	60 (60.6%)	18 (50.0%)	12 (70.6%)	30 (65.2%)	
Humerus	31 (31.3%)	16 (44.4%)	3 (17.6%)	12 (26.1%)	
Tibia	7 (7.1%)	2 (5.6%)	2 (11.8%)	3 (6.5%)	
Radius	1 (1.0%)	0 (0.0%)	0 (0.0%)	1 (2.2%)	
Surgical management					<0.001
Nonoperative	30 (30.3%)	26 (72.2%)	4 (23.5%)	0 (0.0%)	
Intralesional	7 (7.1%)	4 (11.1%)	3 (17.6%)	0 (0.0%)	
Resection	61 (61.6%)	6 (16.7%)	10 (58.8%)	45 (97.8%)	
Amputation	1 (1.0%)	0 (0.0%)	0 (0.0%)	1 (2.2%)	
Maximum imaging dimension, cm	6.90 (3.65–11.20)	3.25 (1.90–5.78)	5.20 (4.00–9.00)	11.00 (7.05–13.50)	<0.001
Documented pain	85 (85.9%)	27 (75.0%)	14 (82.4%)	44 (95.7%)	0.018

Values are n (%) or median (interquartile range), using the full column denominators. Age refers to age at imaging; maximum lesion dimension was measured on imaging. Age, sex, size, and pain status were complete. Pain refers to the documented presence or absence of pain at presentation to the tertiary cancer center, but incidental discovery was not systematically recorded and cannot be inferred from absence of pain. Surgical management uses the recorded surgical-intent field. Continuous comparisons used Kruskal–Wallis; sex and pain used Fisher–Freeman–Halton exact tests, and larger categorical tables used a 99,999-sample Monte Carlo approximation. BACTIP = Birmingham Atypical Cartilage Tumor Imaging Protocol; DDCS = dedifferentiated chondrosarcoma; RAS = Radiological Aggressiveness Score.

**Table 2:** RAS status and reference diagnosis across BACTIP categories.

BACTIP Category	Total n	RAS ≥ 4	Enchondroma	Grade 1 CS	Grade 2 CS	Grade 3 CS	DDCS
Not meeting referral criteria	36	0 (0.0%)	27 (75.0%)	9 (25.0%)	0 (0.0%)	0 (0.0%)	0 (0.0%)
1A	16	0 (0.0%)	15 (93.8%)	1 (6.2%)	0 (0.0%)	0 (0.0%)	0 (0.0%)
1B	4	0 (0.0%)	3 (75.0%)	1 (25.0%)	0 (0.0%)	0 (0.0%)	0 (0.0%)
2A	8	0 (0.0%)	5 (62.5%)	3 (37.5%)	0 (0.0%)	0 (0.0%)	0 (0.0%)
2B	8	0 (0.0%)	4 (50.0%)	4 (50.0%)	0 (0.0%)	0 (0.0%)	0 (0.0%)
Meeting referral criteria	63	46 (73.0%)	4 (6.3%)	12 (19.0%)	19 (30.2%)	5 (7.9%)	23 (36.5%)
1C	3	0 (0.0%)	2 (66.7%)	1 (33.3%)	0 (0.0%)	0 (0.0%)	0 (0.0%)
2C	7	4 (57.1%)	2 (28.6%)	2 (28.6%)	0 (0.0%)	0 (0.0%)	3 (42.9%)
3	53	42 (79.2%)	0 (0.0%)	9 (17.0%)	19 (35.8%)	5 (9.4%)	20 (37.7%)

Percentages represent the proportion of lesions within each BACTIP category. BACTIP = Birmingham Atypical Cartilage Tumor Imaging Protocol; CS = chondrosarcoma; DDCS = dedifferentiated chondrosarcoma; RAS = Radiological Aggressiveness Score.

**Table 3:** (**A**) Two-by-two classification counts for resection-grade disease. (**B**) Diagnostic performance for resection-grade disease in the selected cohort. (**C**) Sensitivity by reference diagnosis.

**(A)**					
**Classification**	**TP**	**FP**	**TN**	**FN**	**Total**
BACTIP referral criteria	47	16	36	0	99
Sequential BACTIP-RAS	43	3	49	4	99
RAS alone	43	3	49	4	99
BACTIP excluding DDCS	24	16	36	0	76
Sequential or RAS alone excluding DDCS	20	3	49	4	76
**(B)**					
**Classification**	**Sensitivity, % (95% CI)**	**Specificity, % (95% CI)**	**PPV, % (95% CI)**	**NPV, % (95% CI)**	**Accuracy, % (95% CI)**
BACTIP referral criteria (n = 99)	100.0 (92.4–100.0)	69.2 (55.7–80.1)	74.6 (62.7–83.7)	100.0 (90.4–100.0)	83.8 (75.3–89.8)
Sequential BACTIP-RAS (n = 99)	91.5 (80.1–96.6)	94.2 (84.4–98.0)	93.5 (82.5–97.8)	92.5 (82.1–97.0)	92.9 (86.1–96.5)
RAS alone (n = 99)	91.5 (80.1–96.6)	94.2 (84.4–98.0)	93.5 (82.5–97.8)	92.5 (82.1–97.0)	92.9 (86.1–96.5)
BACTIP excluding DDCS (n = 76)	100.0 (86.2–100.0)	69.2 (55.7–80.1)	60.0 (44.6–73.7)	100.0 (90.4–100.0)	78.9 (68.5–86.6)
Sequential or RAS alone excluding DDCS (n = 76)	83.3 (64.1–93.3)	94.2 (84.4–98.0)	87.0 (67.9–95.5)	92.5 (82.1–97.0)	90.8 (82.2–95.5)
**(C)**					
**Reference Diagnosis**	**BACTIP Positive/Total**	**Sensitivity % (95% CI)**	**Sequential or RAS Positive/Total**	**Sensitivity % (95% CI)**	
Grade 2	19/19	100.0 (83.2–100.0)	15/19	78.9 (56.7–91.5)	
Grade 3	5/5	100.0 (56.6–100.0)	5/5	100.0 (56.6–100.0)	
Dedifferentiated	23/23	100.0 (85.7–100.0)	23/23	100.0 (85.7–100.0)	

(A) TP = true positive; FP = false positive; TN = true negative; FN = false negative. The reference-positive group comprises grade 2, grade 3, and dedifferentiated chondrosarcoma (DDCS), or grade 2/3 after DDCS exclusion. The reference-negative group comprises enchondroma and grade 1 chondrosarcoma. (B) Values are percentages (95% Wilson confidence intervals). Positive BACTIP classification comprises categories 1C, 2C, and 3; positive sequential classification requires BACTIP referral criteria and RAS ≥ 4. RAS alone and sequential classification identify identical patients in this cohort. All estimates use the complete indicated cohort, not only patients meeting referral criteria. CI = confidence interval; NPV = negative predictive value; PPV = positive predictive value; DDCS = dedifferentiated chondrosarcoma. (C) Wilson confidence intervals are shown. These are within-grade sensitivities, not grade-versus-all-other-diagnoses classification analyses. Specificity is not defined within a subgroup containing only reference-positive lesions.

**Table 4:** Descriptive oncologic outcomes by sequential BACTIP-RAS classification.

Outcome	All Patients (n = 99)	Not Meeting Referral Criteria (n = 36)	Meeting Referral Criteria, RAS < 4 (n = 17)	Meeting Referral Criteria, RAS ≥ 4 (n = 46)	Time Frame
Final status—ANED	65 (65.7%)	35 (97.2%)	14 (82.4%)	16 (34.8%)	Last recorded contact
Final status—AWD	1 (1.0%)	0 (0.0%)	0 (0.0%)	1 (2.2%)	Last recorded contact
Final status—DOD	21 (21.2%)	0 (0.0%)	1 (5.9%)	20 (43.5%)	By last recorded contact
Final status—death from another cause	12 (12.1%)	1 (2.8%)	2 (11.8%)	9 (19.6%)	By last recorded status
Metastases at diagnosis	3 (3.0%)	0 (0.0%)	0 (0.0%)	3 (6.5%)	Baseline
Local recurrence	7 (7.1%)	0 (0.0%)	0 (0.0%)	7 (15.2%)	During available follow-up
New metastases	19/96 (19.8%)	0/36 (0.0%)	1/17 (5.9%)	18/43 (41.9%)	After baseline, among initially nonmetastatic patients
Reverse Kaplan–Meier follow-up, days (IQR)	1777 (979–3939)	1162 (833–2367)	3312 (1314–4718)	2301 (1436–3914)	From surgery or initial imaging

Values are n (%) or reverse Kaplan–Meier median follow-up (interquartile range). Final status, baseline metastases and local recurrence use full column denominators (99/36/17/46). New metastases use patients without metastases at presentation (96/36/17/43), as indicated in the cells. Event percentages are observed proportions, not cumulative-incidence estimates or adjusted risks. Baseline metastases refer to presentation, and recurrence and new metastases refer to available follow-up from surgery or initial imaging. Final status refers to last recorded status. All 12 other-cause deaths were unrelated to the tumor. Reverse Kaplan–Meier follow-up treats deaths as censored and observation ending alive as an event, and quartiles are follow-up-distribution quantiles, not confidence limits. ANED = alive with no evidence of disease; AWD = alive with disease; DOD = died of disease.

## Data Availability

The data are not publicly available because they contain potentially identifying health information and are subject to institutional privacy restrictions. De-identified data may be available from the corresponding author upon reasonable request and subject to institutional approval.

## Supplementary Materials

The following supporting information can be downloaded at: https://www.mdpi.com/article/10.3390/diagnostics16213583/s1, Table S1: Diagnostic sensitivity analyses; Table S2: RAS scoring for false negative grade 2 tumors; Table S3: Reference standards and follow-up; Table S4: Five-year Kaplan–Meier estimates. Table S5: Exploratory temporal comparisons; Table S6: Exploratory age- and sex-adjusted models.

## Abbreviations

The following abbreviations are used in this manuscript:

BACTIP	Birmingham Atypical Cartilage Tumor Imaging Protocol
RAS	Radiological Aggressiveness Score
ANED	Alive with no evidence of disease
AWD	Alive with disease
DOD	Died of disease
BTI-RADS	Bone Tumor Imaging Reporting and Data System
OT-RADS	Osseous Tumor Reporting and Data System
Bone-RADS	Bone Reporting and Data System
REST	Radiological Evaluation Score for Bone Tumors
CS	Chondrosarcoma
DDCS	Dedifferentiated chondrosarcoma
PPV	Positive predictive value
NPV	Negative predictive value
IQR	Interquartile range
CI	Confidence interval
MRI	Magnetic Resonance Imaging
CT	Computed Tomography
ACT	Atypical cartilaginous tumor
