# Supplementary material for: Sequential Application of the Birmingham Atypical Cartilage Tumor Imaging Protocol and Radiological Aggressiveness Score in Long-Bone Intramedullary Cartilage Tumors: A Retrospective Cohort Study
Source: Diagnostics (Basel). 2026 Oct 30;16(21):3583. doi: 10.3390/diagnostics16213583 (PMC13761742; doi:10.3390/diagnostics16213583)
Supplement: Supplementary file 1 [file diagnostics-16-03583-s001.zip › diagnostics-4629951-supplementary.pdf]

BACTIP RAS supplementary tables

Additional diagnostic and survival analyses for the 99-patient cohort. All diagnostic confidence intervals are two-sided 95% Wilson intervals. Referral groups were assigned retrospectively.

Table S1. Diagnostic sensitivity analyses

Table S1A. Histologically confirmed lesions

Classification	N	TP	FP	TN	FN
BACTIP	69	47	12	10	0
Sequential BACTIP–RAS	69	43	3	19	4

Measure	BACTIP	Sequential or RAS alone
Sensitivity	100.0 (92.4–100.0)	91.5 (80.1–96.6)
Specificity	45.5 (26.9–65.3)	86.4 (66.7–95.3)
PPV	79.7 (67.7–88.0)	93.5 (82.5–97.8)
NPV	100.0 (72.2–100.0)	82.6 (62.9–93.0)
Accuracy	82.6 (72.0–89.8)	89.9 (80.5–95.0)

Table S1B. BACTIP original anatomical sites

Classification	N	TP	FP	TN	FN
BACTIP	70	24	13	33	0
Sequential BACTIP–RAS	70	22	2	44	2

Measure	BACTIP	Sequential or RAS alone
Sensitivity	100.0 (86.2–100.0)	91.7 (74.2–97.7)
Specificity	71.7 (57.5–82.7)	95.7 (85.5–98.8)
PPV	64.9 (48.8–78.2)	91.7 (74.2–97.7)
NPV	100.0 (89.6–100.0)	95.7 (85.5–98.8)
Accuracy	81.4 (70.8–88.8)	94.3 (86.2–97.8)

Values are percentages (95% CI). TP/FP/TN/FN denote true/false positive/negative. Histological verification was based on surgical pathology (n=69). The anatomical subset comprises proximal humerus (n=29), distal femur (n=33), proximal tibia (n=7), and a distal-femur/proximal-tibia description (n=1). Twenty-one lesions were explicitly outside the original sites and eight had whole-bone descriptions of uncertain segment. RAS alone and sequential classification were identical. Whole-cohort, DDCS-excluded and grade-specific estimates appear in Tables 3A–3C.

Table S2.RAS scoring for false negative grade 2 tumors

Feature	Case 1	Case 2	Case 3	Case 4
Pathologic Fracture	0	0	0	0
Endosteal Scalloping: Circumference	0	0	0	0
Endosteal Scalloping: Length	0	0	1	0
Medullary Expansion	1	1	0	0
Complete Cortical Erosion/Break	0	0	0	0
Periosteal Reaction	1	1	0	1
Cortical Thickening	0	0	0	0
Marrow Oedema (MRI)	0	1	0	1
Soft-Tissue Component (MRI)	0	0	1	0
RAS total	2	3	2	2
BACTIP category	3	3	3	3
Preoperative biopsy	Not recorded	Grade 1	Grade 1	Grade 1

Binary components: 1=present, 0=absent. All four cases had grade 2 surgical histology. Case labels are anonymous and follow the analysis order. BACTIP category 3 also included bone expansion, cortical thickening and periostitis, and it did not require cortical destruction or a soft-tissue mass. The small number of cases does not establish which features independently predict underclassification.

Table S3. Reference standards and follow up

Measure	All	Non-referral	Referral RAS <4	Referral RAS ≥4
Patients	99	36	17	46
Surgical histopathology	69	10	13	46
Biopsy-only reference diagnosis	0	0	0	0
Clinical/imaging follow-up reference	30	26	4	0
Reverse KM median follow-up, days	1777	1162	3312	2301
Reverse KM follow-up IQR, days	979–3939	833–2367	1314–4718	1436–3914
Observed follow-up <2 years	32	8	2	22
Death from any cause	33	1	3	29

All 30 nonsurgical patients had at least one year of clinical and imaging follow-up and none underwent biopsy. Treating oncologists and radiologists established diagnoses from the clinical and radiological presentation. Retrospective imaging stability meant unchanged BACTIP category on serial MRI and radiographs. Imaging follow-up was complete for the included patients: median 1221 days (IQR 850.5–1393; range 371–5972), or 3.34 years (IQR 2.33–3.81). This does not establish attendance at every scheduled visit or ascertainment after last contact. Reverse Kaplan–Meier median follow-up for nonsurgical patients was 1328 days (IQR 845–2367); five had observed follow-up shorter than two years and none died. Follow-up began at surgery for operated patients and initial imaging for nonsurgical patients. Reverse Kaplan–Meier treats deaths as censored and follow-up ending alive as events. Short observed follow-up may reflect death or early last contact and is not synonymous with loss to follow-up.

Table S4. Five year Kaplan Meier estimates

Table S4A. Recurrence-free survival

Group	N	Events†	At risk at 5 y	Estimate %	95% CI
Non-referral	36	0	12	100.0	Not estimable*
Referral / RAS <4	17	1	8	92.3	56.6–98.9
Referral / RAS ≥4	43	21	13	49.0	32.6–63.5

Table S4B. Metastasis-free survival

Group	N	Events†	At risk at 5 y	Estimate %	95% CI
Non-referral	36	0	12	100.0	Not estimable*
Referral / RAS <4	17	1	8	92.3	56.6–98.9
Referral / RAS ≥4	43	18	13	56.6	39.6–70.5

Table S4C. Disease-specific survival

Group	N	Events†	At risk at 5 y	Estimate %	95% CI
Non-referral	36	0	12	100.0	Not estimable*
Referral / RAS <4	17	1	8	92.3	56.6–98.9
Referral / RAS ≥4	46	20	13	54.0	37.5–67.8

*No endpoint events occurred in the non-referral group. A noninformative 100–100% Greenwood interval is not presented; this does not establish zero underlying risk. †Events are totals over all available follow-up. CIs use Greenwood variance and log–log transformation. Baseline-metastatic patients were excluded from recurrence-free and metastasis-free analyses. All global three-group log-rank tests had 2 df: recurrence-free $\chi^2=32.49$, $p=8.82\times10^{-8}$; metastasis-free $\chi^2=25.98$, $p=2.28\times10^{-6}$; disease-specific $\chi^2=26.17$, $p=2.08\times10^{-6}$. These are exploratory, unadjusted comparisons. Full risk tables and censoring marks are shown in Figure 3.

Table S5. Exploratory temporal comparisons

Table S5A. Patients between 2001–2012

Test	N	TP	FN	TN	FP
BACTIP	46	25	0	13	8
RAS / sequential	46	23	2	20	1

Test	Sensitivity % (95% CI)	Specificity % (95% CI)
BACTIP	100.0 (86.7–100.0)	61.9 (40.9–79.2)
RAS / sequential	92.0 (75.0–97.8)	95.2 (77.3–99.2)

Table S5B. Patients between 2013–May 2025

Test	N	TP	FN	TN	FP
BACTIP	53	22	0	23	8
RAS / sequential	53	20	2	29	2

Test	Sensitivity % (95% CI)	Specificity % (95% CI)
BACTIP	100.0 (85.1–100.0)	74.2 (56.8–86.3)
RAS / sequential	90.9 (72.2–97.5)	93.5 (79.3–98.2)

Periods were assigned by surgery date for operated patients and initial imaging date for nonsurgical patients. Surgery date is a proxy for the imaging era. The periods cover the complete inclusion interval and are not separate sampling frames. For sequential classification/RAS alone, two-sided Fisher exact comparisons of sensitivity and specificity between periods both yielded p=1.00. Similar point estimates do not establish equivalence: subgroup sizes are small, confidence intervals are wide, case mixes differ, and scanner/protocol details were not recorded. All confidence intervals use Wilson scores; thresholds were unchanged.

Table S6. Exploratory age and sex adjusted models

Table S6A. Odds of resection-grade disease by age and sex

Predictor	Adjusted OR	Profile 95% CI	Penalized LR p
RAS ≥4 versus <4	106.59	27.90–595.31	<0.001
Age per 10 years	1.30	0.68–2.28	0.399
Male versus female	4.42	1.01–25.09	0.048

Outcome was resection-grade disease (47/99). All 99 patients had complete age at imaging and sex data. RAS and sequential positivity were identical and were not included together. Firth bias reduction was used for the strong association and small cell counts. Intervals are profile penalized-likelihood intervals and tests are penalized likelihood-ratio tests. The sex estimate is imprecise and exploratory. These results do not establish etiologic associations, independent prognostic value, demographic-invariant diagnostic performance, or a validated prediction model. No multiplicity correction was applied.

Table S6B. Conventional maximum likelihood sensitivity check

Predictor	Adjusted OR	Wald 95% CI
RAS ≥4	182.95	32.53–1028.93
Age per10	1.28	0.68–2.41
Male	5.61	1.00–31.39

The conventional estimates illustrate the same direction of association but remain very imprecise. The Firth model is the primary exploratory adjusted analysis. OR = odds ratio; CI = confidence interval; LR = likelihood ratio.
